# The Connectivity Fingerprint of the Human Frontal Cortex, Subthalamic Nucleus, and Striatum

**DOI:** 10.3389/fnana.2018.00060

**Published:** 2018-07-19

**Authors:** Bethany R. Isaacs, Birte U. Forstmann, Yasin Temel, Max C. Keuken

**Affiliations:** ^1^Integrative Model-Based Cognitive Neuroscience Research Unit, University of Amsterdam, Amsterdam, Netherlands; ^2^Department of Neurosurgery, Maastricht University Medical Center, Maastricht, Netherlands; ^3^Department of Neuroscience, School for Mental Health and Neuroscience, Maastricht University, Maastricht, Netherlands

**Keywords:** 7T MRI, basal ganglia, connectivity, DWI, RS-fMRI

## Abstract

Within the cortico basal ganglia (BG)–thalamic network, the direct and indirect pathways comprise of projections from the cortex to the striatum (STR), whereas the hyperdirect pathway(s) consist of cortical projections toward the subthalamic nucleus (STN). Each pathway possesses a functionally distinct role for action selection. The current study quantified and compared the structural connectivity between 17 distinct cortical areas with the STN and STR using 7 Tesla diffusion weighted magnetic resonance imaging (dMRI) and resting-state functional MRI (rs-fMRI) in healthy young subjects. The selection of these cortical areas was based on a literature search focusing on animal tracer studies. The results indicate that, relative to other cortical areas, both the STN and STR showed markedly weaker structural connections to areas assumed to be essential for action inhibition such as the inferior frontal cortex pars opercularis. Additionally, the cortical connectivity fingerprint of the STN and STR indicated relatively strong connections to areas related to voluntary motor initiation such as the cingulate motor area and supplementary motor area. Overall the results indicated that the cortical–STN connections were sparser compared to the STR. There were two notable exceptions, namely for the orbitofrontal cortex and ventral medial prefrontal cortex, where a higher tract strength was found for the STN. These two areas are thought to be involved in reward processing and action bias.

## Introduction

The basal ganglia (BG) collectively refer to a group of interconnected subcortical nuclei. The main BG components are the caudate and putamen, which together form the striatum (STR), the internal and external segments of the globus pallidus (GPi and GPe, respectively), the substantia nigra, and the subthalamic nucleus (STN) (Parent and Hazrati, 1995a). Together with the cortex and the thalamus, these BG nuclei form an integrative network consisting of a number of loops involved in a wide range of cognitive, limbic, and motor functions (Albin et al., 1989; Alexander and Crutcher, 1990; Alexander et al., 1990; Temel et al., 2005; Haber and Calzavara, 2009). Each circuit is characterized by three functionally distinct pathways: direct, indirect, and hyperdirect. Anatomically, the direct and indirect pathways entail projections from cortex to the STR, while the hyperdirect pathway bypasses the STR and projects from cortex, directly to the STN (Nambu et al., 2002b; Jahanshahi et al., 2015).

Therefore, both the STN and STR are considered as crucial input structures to the BG and are essential for both optimal and flexible adaptive motor control and action selection, which may arise from a number of scenarios, from goal-directed behaviors to habitual responses (Alexander and Crutcher, 1990; Nambu et al., 2002b; Bogacz and Gurney, 2007; Ding and Gold, 2013). Animal literature indicates that the majority of the cortical input to BG arises from prefrontal and primary motor cortices (Parent and Hazrati, 1995a,b). However, it is generally accepted that frontal connections monosynaptically connecting to the STN are more sparse in comparison to those connecting directly to the STR (Frankle et al., 2006).

To the best of our knowledge, a quantitative comparison of the connectivity profiles between the frontal cortex and the STN and STR in humans has yet to be conducted. There have been numerous studies quantifying the diffusion weighted and resting-state functional MRI (rs-fMRI) connectivity patterns of the cortex, STN, and STR separately (e.g., Di Martino et al., 2008; Draganski et al., 2008; Brunenberg et al., 2012; Lambert et al., 2012; Morris et al., 2016; Cacciola et al., 2017; Anteraper et al., 2018). Though many studies use lower MRI field strengths, where the STN is notoriously difficult to visualize due to its small size and high iron content (Cho et al., 2010; de Hollander et al., 2015, 2017; Forstmann et al., 2017). To compliment the previous connectivity studies, we set out to characterize the connectivity fingerprint of the fontal cortex with the STN and STR in healthy young subjects using ultra-high field 7 Tesla (T) dMRI and rs-fMRI data.

## Materials and Methods

### Participants

Sixteen healthy participants (9 female, age range = 19–28, mean age = 23.13, *SD* = 2.47) were scanned. All participants reported normal or corrected to normal vision and were right-handed, as confirmed by the Edinburgh Inventory (Oldfield, 1971). None of the participants had a history of neurological disorder or currently suffered from psychiatric disorders as indicated by self-report and structured clinical interview. The study was approved by the local ethical committee at the Max Planck Institute for Human Brain and Cognitive Sciences in Leipzig, Germany. Written informed consent was acquired and participants received a monetary reward for participation.

### MRI Sequences

#### Structural Scans

The structural data were obtained from a 7T whole body Siemens MAGNETOM using a 24 channel Nova head coil (NOVA Medical Inc., Wilmington, MA, United States) during two sessions. The first session consisted of a whole-brain MP2RAGE (Marques et al., 2010), a MP2RAGE covering a smaller slab, and a multi-echo 3D FLASH slab (Haase et al., 1986). Whole-brain MP2RAGE scans were collected with the following parameters: 240 sagittal slices, acquisition time (TA) of 10:57 min, repetition time (TR) = 5000 ms, echo time (TE) = 2.45 ms, inversion times (TI1) = 900 ms/(TI2) = 2750 ms, flip angle (FA) = 5°/3°, bandwidth (BW) = 250 Hz/Px, and a voxel size of 0.7 mm isotropic. Zoomed MP2RAGE slab images were acquired to facilitate the registration of FLASH images to whole-brain MP2RAGE images which consisted of 128 sagittal slices, with a TA of 9:07 min, TR = 5000 ms, TE = 3.71 ms, TI1 = 900 ms/TI2 = 2750 ms, FA = 5°/3°, BW = 240 Hz/Px, and 0.6 mm isotropic voxel size. Zoomed FLASH slab images consisted of 128 axial slices covering the midbrain with an TA of 17:18 min, TR = 41 ms, TE = 11.22 ms/20.39 ms/29.57 ms, FA = 14°, BW = 160 Hz/Px, and 0.5 mm isotropic voxel size. For the exact acquisition parameters and the raw data see Forstmann et al. (2014).

#### Diffusion Weighted Imaging

In a second structural scan session, DWI was acquired with a spin echo planar imaging sequence (Heidemann et al., 2010). A total of 100 axial slices were acquired with a TA of 54:16 min, TR = 11.3 s, TE = 67 ms, and voxel size = 1.0 mm isotropic and GRAPPA acceleration factor 3. Diffusion weighting was isotropically distributed along 60 directions with a *b* value of 1000 s/mm^2^, AV = 4, and 7 diffusion weighted images to every *B*_0_ image.

#### Resting-State Functional MRI

Finally, in a third MRI session, rs-fMRI was acquired using a 2D EPI sequence. A total of 76 slices were acquired interleaved in transversal direction, with a TA of 5:16 min, TR = 3330 ms, TE = 18 ms, voxel size = 1.5 mm isotropic, phase encoding A > P, GRAPPA acceleration factor 3, BW = 1086 Hz/Px, and echo spacing = 1.03 ms. To correct for distortions, a GRE field map with 57 slices was acquired in transversal direction with a TA of 4:53 min, TR = 1500, TE1 = 6.00 ms, TE2 = 7.02 ms, voxel size = 2.0 mm isotropic, FA = 68°, phase encoding A > P, and BW = 259 Hz/Px.

### Region of Interest (ROI) Definition

#### Subcortical Masks

The STN and STR masks have been previously described in Keuken et al. (2014). The STN was parcellated using the multi-echo FLASH, whereas the STR was parcellated using the MP2RAGE slab. In short, both the STN and STR as a whole were manually parcellated by two independent researchers using FSLview (version 4.1.4.; Jenkinson et al., 2012). The STR was not subdivided into its anatomical subdivisions due to the challenges associated with identifying the border between the caudate nucleus, the putamen, and the fundus striati (Neto et al., 2008; Keuken et al., 2014). Only voxels identified by both raters as belonging to the structure were included for further analyses. Given the size of the STN in relation to the resolution of the dMRI and rs-fMRI we decided to only focus on the connectivity profile of the two subcortical structures as a whole and not investigate any topographical organization within the given structures. For more information regarding the parcellation protocol see Keuken et al. (2014, 2017).

#### Cortical Masks

Instead of testing the connectivity of the STN and STR with the entire cortex, we selected a number of cortical areas that have been identified in non-human primate (NHP) tracer studies as connecting to both the STN and STR. These connections were selected by conducting an empirical literature search using the PubMed database^1^. The employed keywords included: “subthalamic nucleus,” “striatum,” “macaque,” “monkey,” “histological,” “tracer(s),” and “connection(s)” and was published in English. All abstracts and resulting full text articles were read by two researchers (BI and MK).

**FIGURE 1:**
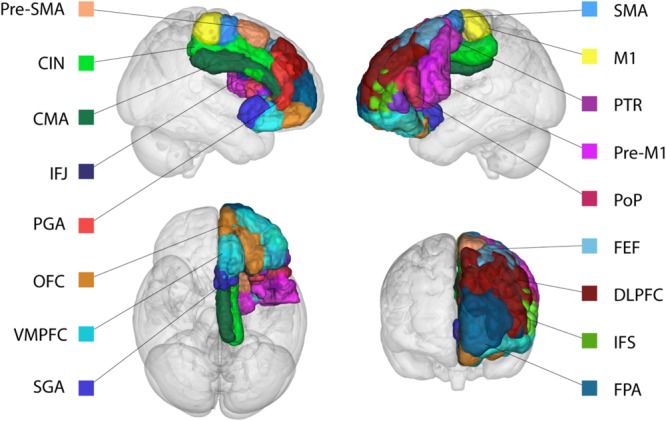
Representation of the cortical region of interests (ROIs). Based on the literature search, all areas have a non-human primate homolog and structurally connect to both the STN and STR in non-human primates. For visualization purposes only the ROIs in the left hemispheres are displayed. See **Table 1** for abbreviations.

Since a large number of studies used different nomenclature to refer to the same or similar brain regions (e.g., Brodmann, Walkers, Vogts, and “own labeling system”) we summarized these studies into a single cortical area using the anatomical description of the original study. The human homolog of each cortical area was then identified in standard MNI-space using a number of comparative anatomical atlases that are based on both human and NHPs (Sallet et al., 2013; Neubert et al., 2014, 2015) as implemented in FSL. See **Table 1** for the cortical areas identified in the literature search and **Figure 1** for a visualization of the cortical ROI’s in MNI-space.

**Table 1:** Cortical areas that connect both to the STR and STN based on tracer studies in non-human primates.

Cortical ROIs	Tracer studies	Cortical masks and corresponding atlas
(1) Primary motor cortex (M1)	Petras, 1968; Kemp and Powell, 1970; Künzle, 1975, 1977; Monakow et al., 1979; Liles and Updyke, 1985; Selemon and Goldman-Rakic, 1985; Nambu et al., 1996, 1997; Takada et al., 1998b, 2001; Tokuno et al., 1999; McFarland and Haber, 2000; Miyachi et al., 2006; Haynes and Haber, 2013	M1 (Neubert et al., 2015)
(2) Pre-motor cortex (pre-M1)	Petras, 1968; Kemp and Powell, 1970; Akert and Künzle, 1978; Monakow et al., 1979; Miyata and Sasaki, 1984; Liles and Updyke, 1985; Selemon and Goldman-Rakic, 1985; Nambu et al., 1997, 2002a; Takada et al., 1998a; McFarland and Haber, 2000; Tachibana et al., 2004; Akkal et al., 2007; Calzavara et al., 2007; Haynes and Haber, 2013	6v, 6r (Neubert et al., 2014), PMd, and PMv (Sallet et al., 2013)
(3) Supplementary motor area (SMA)	Petras, 1968; Kemp and Powell, 1971; Inase et al., 1999; McFarland and Haber, 2000	SMA and pre-SMA (Neubert et al., 2015)
(4) Pre-supplementary motor area (pre-SMA)	Parthasarathy et al., 1992; Nambu et al., 1996, 1997; Takada et al., 1996, 1998a, 2001; Inase et al., 1999; McFarland and Haber, 2000	
(5) Frontal eye fields (FEF)	Künzle and Akert, 1977; Monakow et al., 1979; Stanton et al., 1988; Parthasarathy et al., 1992; Ferry et al., 2000; Calzavara et al., 2007; Borra et al., 2013	8A and 8B (Sallet et al., 2013)
(6) Dorsolateral prefrontal cortex (DLPFC)	Kemp and Powell, 1970; Goldman and Nauta, 1977; Akert and Künzle, 1978; Selemon and Goldman-Rakic, 1985, 1988; Uylings and Van Eden, 1990; Parthasarathy et al., 1992; Ferry et al., 2000; Frankle et al., 2006; Calzavara et al., 2007; Borra et al., 2013; Haynes and Haber, 2013	46, 9, 9/46d, and 9/46v (Sallet et al., 2013)
(7) Frontopolar area (FPA)	Kemp and Powell, 1970; Yeterian and Van Hoesen, 1978; Selemon and Goldman-Rakic, 1985, 1988; Ferry et al., 2000; Haynes and Haber, 2013	10 (Sallet et al., 2013) and FPm, FPl (Neubert et al., 2014)
(8) Ventromedial and lateral prefrontal cortex (VMPFC)	Haber et al., 1995; Ferry et al., 2000; Frankle et al., 2006; Haynes and Haber, 2013	47o, 47m, and 14m (Neubert et al., 2015)
(9) Orbitofrontal cortex (OFC)	Kemp and Powell, 1970; Selemon and Goldman-Rakic, 1985; Haber et al., 1995; Ferry et al., 2000; Frankle et al., 2006; Haynes and Haber, 2013	11 and 11m (Neubert et al., 2015)
(10) Inferior frontal sulcus (IFS)	Yeterian and Van Hoesen, 1978; Ferry et al., 2000; Borra et al., 2013	IFS and IFJ (Neubert et al., 2014)
(11) Inferior frontal junction (IFJ)		
(12) Inferior frontal gyrus pars opercularis (POP)	Yeterian and Van Hoesen, 1978; Ferry et al., 2000; Borra et al., 2013	POP: 44d, 44v, and PTR: 45 (Neubert et al., 2014)
(13) Inferior frontal gyrus pars triangularis (PTR)		
(14) Cingulate cortex (CIN)	Yeterian and Van Hoesen, 1978; Selemon and Goldman-Rakic, 1985; Ferry et al., 2000; McFarland and Haber, 2000; Frankle et al., 2006; Calzavara et al., 2007; Haynes and Haber, 2013	23ab and 24 (Neubert et al., 2015)
(15) Cingulate motor area (CMA)	Ferry et al., 2000; McFarland and Haber, 2000; Takada et al., 2001	CCZ, RCZa, and RCZp (Neubert et al., 2015)
(16) Perigenual area (PGA)	Ferry et al., 2000; Freedman et al., 2000; Frankle et al., 2006	PGA: 32pl, 32d and SGA: 25 (Neubert et al., 2015)
(17) Subgenual area (SGA)		

*The separate cortical masks from Sallet et al., (2013) and Neubert et al., (2014, 2015) were extracted from FSL, combined in a single region if relevant, binarized, and used as a cortical ROI.*

### MRI Registration

#### Subcortical Masks

The average FLASH volume of the three TE’s was linearly registered to the MP2RAGE whole-brain second inversion volume using a mutual information function, trilinear interpolation, and 6 degrees of freedom (DoF) in FLIRT (FSL 5.0.9). The MP2RAGE slab image was linearly registered to the MP2RAGE whole-brain UNI volume using a correlation cost function, trilinear interpolation, and 6 DoF in FLIRT. The MP2RAGE whole-brain was registered to the average rs-fMRI volume using mutual information cost function, trilinear interpolation, and 6 DoF. The MP2RAGE whole-brain was registered to the *B*_0_ volume using correlation cost function, trilinear interpolation, and 7 DoF (6 DoF did not result in a reasonable registration). All images were skull stripped before registration using BET (Smith, 2002). All registrations were visually inspected. The resulting transformation matrices from the slab to whole-brain and whole-brain to either the *B*_0_ or average rs-fMRI were concatenated and used to transform the STN and STR masks to the either DWI or rs-fMRI space using nearest neighbor interpolation.

#### Cortical Masks

The skull stripped 1 mm MNI template was linearly registered to the MP2RAGE whole-brain UNI volume using a correlation cost function, trilinear interpolation, and 12 DoF in FLIRT. The resulting transformation matrix were concatenated with the transformation matrix of the whole-brain to either the *B*_0_ or average rs-fMRI and used to transform the cortical masks to the either DWI or rs-fMRI space using nearest neighbor interpolation.

### DWI Processing

Diffusion weighted image pre-processing and all subsequent analyses were conducted using FSL (version 5.0.10). The four runs were concatenated and the data were corrected for eddy currents and motion. A single volume without diffusion weighting (*B*_0_) was extracted from the DWI volume and used to create a brain-mask using BET (Smith, 2002). BedpostX (Behrens et al., 2003) was then run on the pre-processed data to estimate the voxel-wise diffusion parameter distributions. Then probabilistic tractography was used to estimate the tract strength between the cortical and subcortical regions. This was done in both directions using a midline exclusion mask, 5000 samples, a curvature threshold of 0.2, and a distance correction was used to correct for differences in cortical–subcortical distance (Azadbakht et al., 2015). See **Table 2** for the average path cortical–subcortical length for the STN and STR separately. The tractography analysis resulted in an image which, for each voxel in the seed mask, contains the number of samples reaching the target mask.

**Table 2:** Summary statistics of the tract lengths between the cortical areas and the STN and STR averaged over hemispheres.

	STN		STR		
	Mean	*SD*	Mean	*SD*	BF_10_
(1) Primary motor cortex (M1)	104.3	22.76	107.3	20.88	0.85
(2) Pre-motor cortex (pre-M1)	92.62	7.23	100.32	9.18	781.37
(3) Supplementary motor area (SMA)	96.96	6.36	102.37	5.98	14.82
(4) Pre-supplementary motor area (pre-SMA)	93.45	6.17	107.22	11.29	≥1000
(5) Frontal eye fields (FEF)	95.56	7.51	104.21	10.84	≥1000
(6) Dorsolateral prefrontal cortex (DLPFC)	94.36	9.59	97.92	12.6	1.44
(7) Frontopolar area (FPA)	95.48	11.61	86.59	11.47	≥1000
(8) Ventromedial and lateral prefrontal cortex (VMPFC)	70.62	20.28	45.08	14.02	≥1000
(9) Orbitofrontal cortex (OFC)	61.28	14.68	37.0	11.39	≥1000
(10) Inferior frontal sulcus (IFS)	99.85	8.46	107.13	11.78	45.70
(11) Inferior frontal junction (IFJ)	103.38	10.8	104.81	14.09	0.25
(12) Pars opercularis (POP)	76.97	18.53	95.07	13.55	≥1000
(13) Pars triangularis (PTR)	79.25	17.55	94.35	14.07	≥1000
(14) Cingulate cortex (CIN)	93.77	15.25	92.25	12.15	0.24
(15) Cingulate motor area (CMA)	86.47	6.85	91.14	4.82	33.16
(16) Perigenual area (PGA)	88.86	11.5	87.12	15.47	0.26
(17) Subgenual area (SGA)	44.63	25.48	48.36	11.59	0.30

*The BF_10_ are the resulting Bayes factors from the paired *t*-tests between the STN and STR tracts.*

#### Calculation of Tract Seed Ratio

To remove any spurious connections, the resulting seed images were thresholded so that only voxels which had at least 50 samples were kept. The resulting thresholded masks were divided by the number of samples (*n* = 5000). This creates a ratio between the number of samples that reached the target versus the total number of samples used per voxel. Finally, an average ratio was calculated for a given seed mask which indicates, on average, how many samples ended in the target region. Tract seed ratio was defined here as the average of the two seed ratios that resulted from the seed-to-target tractography and target-to-seed tractography.

#### Calculation of Tract Strength

Tract seed ratio can be informative to show differences in connectivity between regions it does not take differences in volume into account. Therefore, in addition to tract seed ratio we also calculate tract strength. To remove any spurious connections, the resulting seed image was thresholded so that only voxels which had at least 50 samples were kept. The number of non-zero voxels was then divided by the total number of voxels in the seed mask, resulting in a ratio indicating the proportion of seed mask voxels that was probabilistically connected to the target mask. This ratio is relative to the volume of the seed mask and compensates for the volumetric differences between the STN and STR. Tract strength was defined as the average of the two ratios that resulted from the seed-to-target tractography and target-to-seed tractography (Forstmann et al., 2010; Boekel et al., 2017). Tract strength differs from tract seed ratio in two ways: the absolute number of samples is not taken into account but the volumes of the seed masks are. Note that the term tract strength here is used to index a probability density function (PDF), quantifying the ratio of how many streamlines directly and continuously commence from a seed region and terminate at a target area. This PDF is a commonly used measure for inferring the strength of structural white matter tracts (Behrens et al., 2007; Khalsa et al., 2014; van den Bos et al., 2014). While the PDF is a commonly used measure for inferring the strength of white matter tracts, it is not without its limitations. For instance, the “amount” of probability or confidence we have in a tract can be influenced by the distance between two areas. As errors and noise accumulate over time, shorter connections would result in higher tract strengths (Jbabdi and Johansen-Berg, 2011). Therefore we used a distance correction. While the tract strength ratio is normalized for volume and a distance correction was applied, the direct statistical comparisons between the STN and STR should be interpreted with caution.

### rs-fMRI Processing

The rs-fMRI data were corrected for *B*_0_ field inhomogeneities using fsl_prepare_fieldmap and FUGUE as implemented in FSL (version 5.0.9). Subsequently, the rs-fMRI data were motion-corrected using MCFLIRT. The average time series of each cortical and subcortical ROI were correlated using a Pearson correlation, and the correlation coefficient was used for further analyses.

### Statistics

The outlier criteria were three times the interquartile range. All statistics were done using the Bayesian tests implemented in the BayesFactor toolbox (Morey et al., 2014) in R (R Core Team, 2016). The benefit of using Bayesian statistics is that it allows the quantification of evidence for the null hypothesis (H_0_: the STN and STR do not differ in tract strength or rs-fMRI correlation) versus the alternative hypothesis (H_1_: the STN and STR do differ in tract strength or rs-fMR correlation). We will use the labels as proposed by Jeffreys (1961) and adjusted by Wetzels and Wagenmakers (2012) and are shown in **Table 3**. Bayes factors which are larger or equal to 1000 will be noted as ≥1000. The tract strengths and correlation coefficients were compared using a JZS Bayesian mixed effect model with subcortical region and cortical region as independent variables, and participant and hemisphere as random factors with default prior scales as implemented in the BayesFactor toolbox.

**Table 3:** Suggested categories for interpreting the Bayes factors.

Bayes factor BF_10_			Interpretation
	>	100	Decisive evidence for H_1_
30	–	100	Very strong evidence for H_1_
10	–	30	Strong evidence for H_1_
3	–	10	Substantial evidence for H_1_
1	–	3	Anecdotal evidence for H_1_
	1		No evidence
1/3	–	1	Anecdotal evidence for H_0_
1/10	–	1/3	Substantial evidence for H_0_
1/30	–	1/10	Strong evidence for H_0_
1/100	–	1/30	Very strong evidence for H_0_
	<	1/00	Decisive evidence for H_0_

### Open Science

All corresponding analysis scripts can be found on https://osf.io/s46hr/

## Results

### Average Seed Ratio Differences Between the STN and STR

The outlier analysis indicated that for a single tract [STN – orbitofrontal cortex (OFC)] there were four outliers. These data points were removed from any further analysis. The JZS Bayesian mixed effect model revealed that the model with main effects for subcortical structure and cortical structures, as well as an interaction between these two variables, is preferred over the model without the interaction, by a Bayes factor of >1000. Therefore, the data provide decisive evidence that the average number of samples reaching the target is generally higher for the tracts between the STN and cortex than for the STR. Pairwise *post hoc* comparisons between the STN and STR are given in **Table 4**.

**Table 4:** Summary statistics of the average seed ratios for the STN and STR to cortex averaged over hemispheres.

	STN		STR		
	Mean	*SD*	Mean	*SD*	BF_10_
(1) Primary motor cortex (M1)	0.21	0.02	0.18	0.01	≥1000
(2) Pre-motor cortex (pre-M1)	0.18	0.01	0.16	0.01	≥1000
(3) Supplementary motor area (SMA)	0.19	0.01	0.16	0.01	≥1000
(4) Pre-supplementary motor area (pre-SMA)	0.18	0.01	0.16	0.01	≥1000
(5) Frontal eye fields (FEF)	0.19	0.02	0.17	0.02	≥1000
(6) Dorsolateral prefrontal cortex (DLPFC)	0.18	0.01	0.16	0.01	≥1000
(7) Frontopolar area (FPA)	0.18	0.02	0.15	0.01	≥1000
(8) Ventromedial and lateral prefrontal cortex (VMPFC)	0.15	0.02	0.14	0.01	17.56
(9) Orbitofrontal cortex (OFC)	0.15	0.01	0.15	0.01	0.24
(10) Inferior frontal sulcus (IFS)	0.19	0.02	0.17	0.02	≥1000
(11) Inferior frontal junction (IFJ)	0.20	0.02	0.17	0.02	≥1000
(12) Pars opercularis (POP)	0.17	0.02	0.17	0.02	0.19
(13) Pars triangularis (PTR)	0.17	0.02	0.17	0.02	0.19
(14) Cingulate cortex (CIN)	0.19	0.02	0.17	0.01	≥1000
(15) Cingulate motor area (CMA)	0.17	0.01	0.16	0.01	≥1000
(16) Perigenual area (PGA)	0.17	0.02	0.16	0.01	909.18
(17) Subgenual area (SGA)	0.13	0.04	0.14	0.02	0.52

*The BF_10_ are the resulting Bayes factors from the paired *t*-tests between the STN and STR tracts. For illustrative purposes the average seed ratio values in this table are multiplied with 10.*

### Tract Strength Differences Between STN and STR

The outlier analysis indicated that for a single tract [STR – perigenual area (PGA)] there was a single outlier. This data point was removed from any further analysis. The JZS Bayesian mixed effect model revealed that the model with main effects for subcortical structure and cortical structures, as well as an interaction between these two variables, is preferred to the model without the interaction with a Bayes factor of >1000. Therefore, the data provide decisive evidence that the tract strength between the STN and cortex is generally lower than for the STR and the cortex. Note that this was the case even though the absolute number of samples reaching the target was higher for the STN.

**FIGURE 2:**
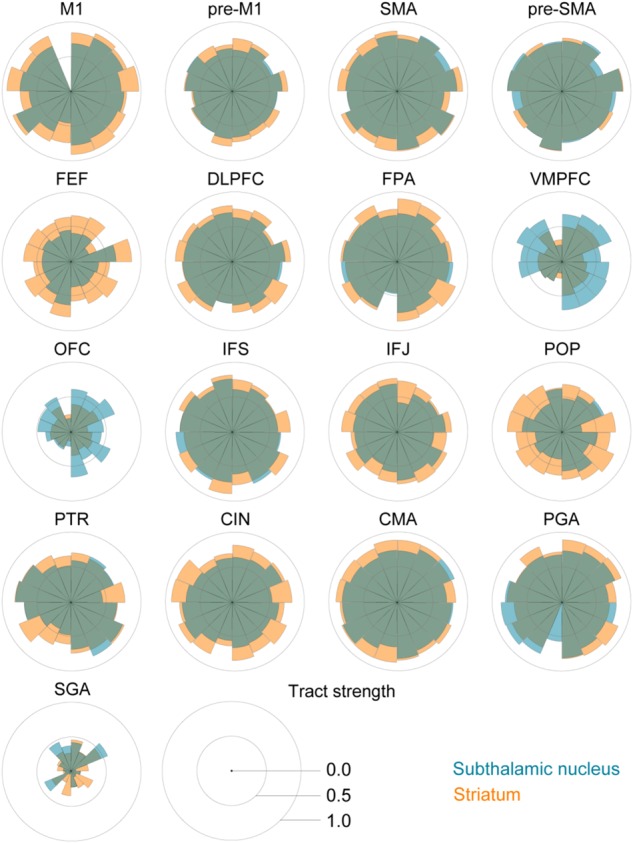
Star plots of the tract strengths between the STN, STR, and the different cortical regions per participant. The STN is color coded using blue, the STR using orange, and both tracts are plotted with 50% opacity. Each segment corresponds to an individual participant.

The *post hoc* comparisons indicated a few exceptions, namely that there was decisive evidence for the STN for higher tract strengths toward the ventromedial and lateral prefrontal cortex (VMPFC) and OFC. There was substantial evidence for the STN and the STR showing similar tract strengths to the pre-supplementary motor area (pre-SMA). There was only anecdotal evidence that tract strengths for the PGA and subgenual area (SGA) were similar between the STN and STR (see **Table 5** for the paired *t*-tests between the STN and STR per tract and **Figure 2**).

**Table 5:** Summary statistics of the tract strengths for the STN and STR to cortex averaged over hemispheres.

	STN		STR		
	Mean	*SD*	Mean	*SD*	BF_10_
(1) Primary motor cortex (M1)	0.71	0.1	0.84	0.09	≥1000
(2) Pre-motor cortex (pre-M1)	0.64	0.06	0.69	0.07	139.30
(3) Supplementary motor area (SMA)	0.77	0.08	0.83	0.07	132.52
(4) Pre-supplementary motor area (pre-SMA)	0.74	0.06	0.73	0.09	0.27
(5) Frontal eye fields (FEF)	0.43	0.2	0.65	0.12	≥1000
(6) Dorsolateral prefrontal cortex (DLPFC)	0.67	0.07	0.76	0.09	≥1000
(7) Frontopolar area (FPA)	0.7	0.09	0.79	0.11	131.73
(8) Ventromedial and lateral prefrontal cortex (VMPFC)	0.57	0.2	0.39	0.12	≥1000
(9) Orbitofrontal cortex (OFC)	0.45	0.18	0.3	0.08	≥1000
(10) Inferior frontal sulcus (IFS)	0.7	0.09	0.76	0.1	10.87
(11) Inferior frontal junction (IFJ)	0.61	0.12	0.73	0.07	≥1000
(12) Pars opercularis (POP)	0.48	0.15	0.7	0.07	≥1000
(13) Pars triangularis (PTR)	0.63	0.18	0.71	0.08	7.0
(14) Cingulate cortex (CIN)	0.64	0.08	0.78	0.08	≥1000
(15) Cingulate motor area (CMA)	0.76	0.07	0.84	0.06	721.13
(16) Perigenual area (PGA)	0.75	0.11	0.78	0.11	0.37
(17) Subgenual area (SGA)	0.22	0.2	0.27	0.11	0.55

*The BF_10_ are the resulting Bayes factors from the paired *t*-tests between the STN and STR tracts.*

The main effect of cortical areas and the interaction indicated that various cortical areas have different tract strengths to the subcortex and that this tract strength varied per cortical area and subcortical structure.

**FIGURE 3:**
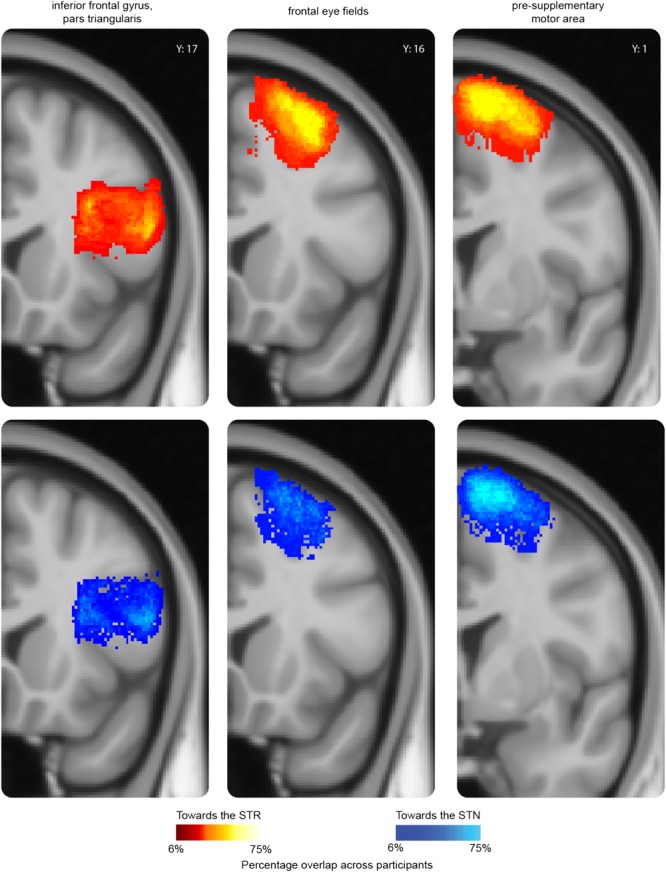
A probability map of three representative cortical regions projecting to the subcortex. For the pars triangularis (PTR), frontal eye fields (FEF), and pre-supplementary motor area (pre-SMA), a probability map was created using the individual thresholded seed masks. The thresholded seed masks only include those voxels which contained at least 50 samples in the tractography analysis. To be able to compare the results across participants, the thresholded seed masks were transformed back into MNI-standard space using the inverted transformation matrices. The color intensity indicates the overlap across participants. In red-yellow the probability map of the thresholded seed masks toward the STR; in blue the probability map of the thresholded seed masks toward the STN. The *y* value corresponds to the MNI coordinate system.

As illustrated in **Figure 1**, the cortical masks used in this study are rather large. It might therefore be the case that the cortical areas projecting to the STN did not overlap with the cortical areas projecting to the STR. We illustrated this by backprojecting the thresholded cortical masks from individual *B*_0_ to MNI standard space where a probabilistic map was created across participants (see **Figure 3**). It seemed that the cortical region projecting to the STR that had the highest overlap across participants is the same region that also projects to the STN.

### Tract Strength Differences for the STN and STR Separately

To test for differences between tracts connecting subcortical areas, i.e., STN and STR, with the cortex, Bayesian paired *t*-tests were used (see **Table 6** for all the resulting Bayes factors). The paired *t*-tests indicated that the STN and the STR shared a number of similarities in their connectivity fingerprint to cortex.

**Table 6:** Paired *t*-tests within the STN and STR tracts.

	M1	Pre-M1	SMA	Pre-SMA	FEF	DLPFC	FPA	VMPFC	OFC	IFS	IFJ	POP	PTR	CIN	CMA	PGA	SGA
M1	–	15.17	18.65	0.46	≥1000	0.67	0.2	133.02	≥1000	0.2	67.37	≥1000	1.56	60	250.31	1.19	≥1000
Pre-M1	15.17	–	≥1000	≥1000	≥1000	2.15	9.66	1.05	≥1000	34.54	0.44	≥1000	0.2	0.19	≥1000	324.43	≥1000
SMA	18.65	≥1000	–	0.38	≥1000	457.49	5.39	≥1000	≥1000	2.93	≥1000	≥1000	97.17	≥1000	0.21	0.25	≥1000
Pre-SMA	0.46	≥1000	0.38	–	≥1000	≥1000	0.83	248.91	≥1000	1.96	≥1000	≥1000	10.66	359.66	0.26	0.19	≥1000
FEF	≥1000	≥1000	≥1000	≥1000	–	≥1000	≥1000	2.2	0.21	≥1000	32.15	0.34	133.78	≥1000	≥1000	≥1000	49.93
DLPFC	0.67	2.15	457.49	≥1000	≥1000	–	1.17	5.25	≥1000	1.62	4.59	≥1000	0.43	0.63	291.21	6.37	≥1000
FPA	0.2	9.66	5.39	0.83	≥1000	1.17	–	140.69	≥1000	0.19	10.67	≥1000	2.51	31.77	18.82	2.09	≥1000
VMPFC	133.02	1.05	≥1000	248.91	2.2	5.25	140.69	–	≥1000	18.65	0.34	1.28	0.9	1.21	≥1000	≥1000	≥1000
OFC	≥1000	≥1000	≥1000	≥1000	0.21	≥1000	≥1000	≥1000	–	≥1000	90.73	0.29	≥1000	≥1000	≥1000	≥1000	≥1000
IFS	0.2	34.54	2.93	1.96	≥1000	1.62	0.19	18.65	≥1000	–	227.44	≥1000	1.44	3.98	2.47	0.58	≥1000
IFJ	67.37	0.44	≥1000	≥1000	32.15	4.59	10.67	0.34	90.73	227.44	–	67.98	0.2	0.3	≥1000	243.21	≥1000
POP	≥1000	≥1000	≥1000	≥1000	0.34	≥1000	≥1000	1.28	0.29	≥1000	67.98	–	883.01	≥1000	≥1000	≥1000	≥1000
PTR	1.56	0.2	97.17	10.66	133.78	0.43	2.51	0.9	≥1000	1.44	0.2	883.01	–	0.2	215.06	13.3	≥1000
CIN	60	0.19	≥1000	359.66	≥1000	0.63	31.77	1.21	≥1000	3.98	0.3	≥1000	0.2	–	≥1000	≥1000	≥1000
CMA	250.31	≥1000	0.21	0.26	≥1000	291.21	18.82	≥1000	≥1000	2.47	≥1000	≥1000	215.06	≥1000	–	0.28	≥1000
PGA	1.19	324.43	0.25	0.19	≥1000	6.37	2.09	≥1000	≥1000	0.58	243.21	≥1000	13.3	≥1000	0.28	–	≥1000
SGA	≥1000	≥1000	≥1000	≥1000	49.93	≥1000	≥1000	≥1000	≥1000	≥1000	≥1000	≥1000	≥1000	≥1000	≥1000	≥1000	–

*The right upper triangle displays the BF_10_ of the paired tests for the STR, while the lower left triangle displays the BF_10_ for the STN pairs. To highlight the use of this table, in gray the BF_10_ for the IFJ indicated lower tract strengths compared to other cortical areas for both the STN (dark gray, lower triangle) and STR (light gray, upper triangle). For display purposes, all BF_10_ larger or equal to 1000 are noted as ≥1000.*

Compared to other cortical areas, the SGA, frontal eye fields (FEF), OFC, and the pars opercularis of the inferior frontal gyrus (POP) had lower tract strengths toward the STN (SGA: 16 out of 16 paired *t*-tests indicated substantial or more evidence in favor of lower tract strengths; FEF: 12 out of 16 paired *t*-tests indicated substantial or more evidence in favor of lower tract strengths; OFC: 13 out of 16 paired *t*-tests indicated substantial or more evidence in favor of lower tract strengths; and POP: 12 out of 16 paired *t*-tests indicated substantial or more evidence in favor of lower tract strengths). Similarly, compared to other cortical areas, the SGA, FEF, OFC, and POP had lower tract strengths toward the STR (SGA: 16 out of 16 paired *t*-tests indicated substantial or more evidence in favor of lower tract strengths; FEF: 12 out of 16 paired *t*-tests indicated substantial or more evidence in favor of lower tract strengths; OFC: 13 out of 16 paired *t*-tests indicated substantial or more evidence in favor of lower tract strengths; and POP: 11 out of 16 paired *t*-tests indicated substantial or more evidence in favor of lower tract strengths).

There were also a number of cortical areas that had relatively higher tract strengths toward both the STN and STR. Compared to the other cortical areas, the SMA, cingulate motor area (CMA), PGA, and pre-SMA had higher tract strengths toward the STN (SMA: 12 out of 16 paired *t*-tests indicated substantial or more evidence in favor of higher tract strengths; CMA: 12 out of 16 paired *t*-tests indicated substantial or more evidence in favor of higher tract strengths; PGA: 10 out of 16 paired *t*-tests indicated substantial or more evidence in favor of higher tract strengths; pre-SMA: 10 out of 16 paired *t*-tests indicated substantial or more evidence in favor of higher tract strengths). Similarly, compared to the other cortical areas, the CMA and SMA had higher tract strengths toward the STR (CMA: 12 out of 16 paired *t*-tests indicated substantial or more evidence in favor of higher tract strengths; SMA: 12 out of 16 paired *t*-tests indicated substantial or more evidence in favor of higher tract strengths).

### rs-fMRI Correlation Differences Between STN and STR

There were no outliers for the rs-fMRI correlations. The JZS Bayesian mixed effect model revealed that the model with main effects for subcortical structures and cortical structures, as well as an interaction between these two variables, is preferred to the model without the interaction with a Bayes factor of 130.74. The data therefore provide decisive evidence that the resting-state BOLD correlation between the STN and cortex is generally lower than for the STR and cortex.

**FIGURE 4:**
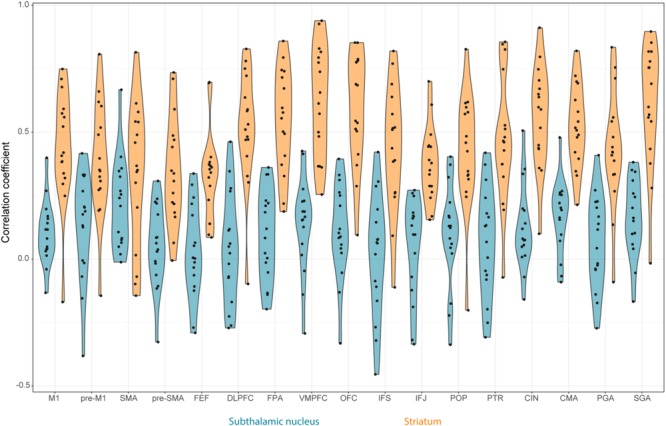
Violin plots of the rs-fMRI correlation between STN, STR, and the different cortical regions per participant. The STN is color coded using blue and the STR using orange. The black circles correspond to the individual participants.

The *post hoc* comparisons indicate that overall the STR has a stronger rs-fMRI correlation to cortical areas compared to the STN (see **Table 7** for the paired *t*-tests between the STN and STR per tract, as well as **Figure 4**). It is unlikely that this difference in rs-fMRI is due to the *T*_2_^∗^ differences because the rs-fMRI sequence used short TE’s optimized for the human subcortex at 7T (Keuken et al., 2015, 2017; de Hollander et al., 2017; Mestres-Missé et al., 2017). It might, however, be the case that there is more physiological noise in and around the STN, resulting in lower rs-fMRI correlations with cortex (Altman and Krzywinski, 2015). Therefore, the direct comparison between the STN and STR as reported in **Tables 3, 4** should be interpreted with caution.

**Table 7:** Summary statistics of rs-fMRI correlation coefficient for the STN and STR to cortex averaged over hemispheres.

	STN		STR		
	Mean	*SD*	Mean	*SD*	BF_10_
(1) Primary motor cortex (M1)	0.12	0.19	0.41	0.43	743.67
(2) Pre-motor cortex (pre-M1)	0.15	0.17	0.44	0.22	>1000
(3) Supplementary motor area (SMA)	0.18	0.18	0.36	0.27	7.26
(4) Pre-supplementary motor area (pre-SMA)	0.04	0.21	0.39	0.22	>1000
(5) Frontal eye fields (FEF)	–0.02	0.23	0.38	0.21	>1000
(6) Dorsolateral prefrontal cortex (DLPFC)	0.04	0.22	0.54	0.21	>1000
(7) Frontopolar area (FPA)	0.08	0.21	0.54	0.21	>1000
(8) Ventromedial and lateral prefrontal cortex (VMPFC)	0.16	0.23	0.63	0.21	>1000
(9) Orbitofrontal cortex (OFC)	0.1	0.24	0.59	0.21	>1000
(10) Inferior frontal sulcus (IFS)	0.07	0.23	0.45	0.23	>1000
(11) Inferior frontal junction (IFJ)	0.07	0.19	0.43	0.16	>1000
(12) Pars opercularis (POP)	0.13	0.18	0.46	0.21	>1000
(13) Pars triangularis (PTR)	0.15	0.2	0.45	0.23	>1000
(14) Cingulate cortex (CIN)	0.13	0.23	0.58	0.21	>1000
(15) Cingulate motor area (CMA)	0.15	0.19	0.55	0.19	>1000
(16) Perigenual area (PGA)	0.1	0.19	0.45	0.24	>1000
(17) Subgenual area (SGA)	0.12	0.2	0.52	0.26	>1000

*The BF_10_ are the resulting Bayes factors from the paired *t*-tests between the STN and STR to cortex pairs.*

### Correlation Differences Within the STN and STR

To test which of the resting-state correlations between the subcortical areas and cortex differed from each other, Bayesian paired *t*-tests for the STN and STR were run separately. The results indicated that the STN had a different resting-state fingerprint than the STR.

Contrary to the tract strengths, most of the rs-fMRI correlations between the STN and cortical ROIs did not differ from each other (see **Table 8** for all the resulting Bayes factors). This was the case for the primary motor area (M1) (11 out of 16 paired *t*-tests indicated substantial or more evidence in favor of no difference); SGA (10 out of 16 paired *t*-tests); pre-M1 (9 out of 16 paired *t*-tests); POP (9 out of 16 paired *t*-tests); the inferior frontal gyrus pars triangularis (PTR; 8 out of 16 paired *t*-tests); CMA (8 out of 16 paired *t*-tests); and OFC (8 out of 16 paired *t*-tests). There was a notable exception for the FEF where compared to the other cortical areas the resting-state correlation with the STN was lower (13 out of 16 paired *t*-tests indicated substantial or more evidence in favor of a lower correlation).

**Table 8:** Paired *t*-tests for the resting-state correlation between the STN, STR, and the cortical ROIs.

	M1	Pre-M1	SMA	Pre-SMA	FEF	DLPFC	FPA	VMPFC	OFC	IFS	IFJ	POP	PTR	CIN	CMA	PGA	SGA
M1	–	0.30	0.43	0.22	0.24	14.0	9.74	579.85	107.47	0.26	0.20	0.41	0.29	436.16	78.01	0.27	0.65
Pre-M1	0.33	–	1.12	0.43	0.53	6.44	6.36	443.40	29.55	0.19	0.22	0.21	0.20	208.54	36.44	0.20	0.41
SMA	0.88	0.27	–	0.21	0.20	40.49	39.79	>1000	259.12	0.56	0.52	1.33	0.64	947.16	>1000	0.90	2.18
Pre-SMA	7.15	44.56	34.68	–	0.20	65.09	55.20	>1000	539.04	0.52	0.37	0.69	0.47	>1000	182.56	0.43	1.15
FEF	34.56	770.56	>1000	4.18	–	>1000	205.76	>1000	>1000	1.51	0.52	1.35	0.80	>1000	>1000	0.69	1.83
DLPFC	1.50	12.87	32	0.19	1.44	–	0.19	73.41	1.50	881.33	199.80	261.95	41.51	1.84	0.19	28.53	0.20
FPA	0.27	0.85	3.33	0.30	5.25	1.27	–	202.08	1.03	27.91	59.36	5.09	7.47	1.09	0.20	12.71	0.20
VMPFC	0.30	0.19	0.20	5.88	72.59	41.10	3.61	–	0.66	>1000	>1000	>1000	>1000	0.61	5.25	>1000	1.10
OFC	0.20	0.33	0.72	0.51	6.54	1.09	0.24	4.69	–	>1000	>1000	>1000	750.84	0.19	0.70	163.26	0.45
IFS	0.31	1.68	2.21	0.21	0.88	0.28	0.20	22.21	0.29	–	0.24	0.20	0.19	668.59	6.72	0.19	0.43
IFJ	0.46	9.82	2.73	0.26	1.90	0.27	0.20	3.65	0.26	0.19	–	0.39	0.25	>1000	870.92	0.25	0.78
POP	0.21	0.28	0.38	6.26	123.63	10.06	0.43	0.28	0.24	0.88	2.64	–	0.18	>1000	133.48	0.19	0.38
PTR	0.25	0.19	0.22	2.83	24.36	11.48	0.81	0.20	0.36	10.85	2.75	0.26	–	281.89	6.38	0.19	0.44
CIN	0.20	0.22	0.43	1.71	48.83	16.92	0.89	0.41	0.35	0.86	0.59	0.19	0.22	–	2.15	>1000	0.34
CMA	0.32	0.19	0.30	15.22	589.96	48.74	1.58	0.20	0.67	1.41	1.81	0.23	0.19	0.32	–	9.46	0.21
PGA	0.21	0.57	1.19	0.45	4.85	1.03	0.23	1.38	0.19	0.35	0.27	0.30	0.71	0.34	0.64	–	0.45
SGA	0.19	0.24	0.36	0.52	3.20	0.65	0.27	0.28	0.21	0.35	0.47	0.19	0.23	0.19	0.23	0.22	–

*The upper triangle displays the BF_10_ of the paired tests for the STR and cortical ROIs, while the lower triangle displays the BF_10_ for the STN pairs. As an example, to highlight the use of this table, in dark gray the BF_10_ for the FEF indicating lower correlations compared to other cortical areas for the STN (lower triangle), while in light gray the BF_10_ for the VMPFC indicating higher correlations for the STR (upper triangle). For display purposes, all BF_10_ larger than 1000 are noted as >1000.*

Contrary to the STN, the rs-fMRI correlations between the STR and the cortical ROIs seemed more heterogeneous. Compared to the other cortical areas, there were a number of regions which had a higher rs-fMRI correlation with the STR such as the ventral medial prefrontal cortex (VMPFC; 13 out of 16 paired *t*-tests indicated substantial or more evidence in favor of a higher correlation); OFC (10 out of 16 paired *t*-tests indicated substantial or more evidence in favor of a higher correlation); and the cingulate cortex (CIN; 10 out of 16 paired *t*-tests indicated substantial or more evidence in favor of a higher correlation).

## Discussion

This study set out to investigate the connectivity fingerprint of the STN and STR with the cortex using diffusion and rs-fMRI. The tract strengths indicate that for most cortical areas tested, the STR exhibits relatively higher tract strengths than the STN. It is unlikely that the lower tract strength for the STN was due to higher noise in the tractography as the absolute seed ratios were actually higher for the STN. For the rs-fMRI data, the correlations between the cortical ROI’s and the STR were also consistently found to be higher than those for the STN. This finding is in line with the previous literature that notes that while the STN and STR are indeed directly connected to similar cortical areas, STN connections are more sparsely present (Alexander et al., 1986; Albin et al., 1989; Frankle et al., 2006).

There were, however, two notable exceptions for tract strengths. Namely for the OFC and VMPFC, where a higher tract strength was found for the STN relative to the STR. The OFC and VMPFC are two cortical regions thought to be essential for reward processing, choice bias, and mood (Hollerman et al., 2000; Haber and Knutson, 2009; Mulder et al., 2013; Lim et al., 2015). Reward-oriented behaviors require many mental processes, including motor, sensory, learning, memory, cognitive, executive, decision-making, motivational, and emotional functions (Crocker et al., 2013). Given the multifaceted and complex nature of limbic processes, having strong connections to the STN might be explained as a direct modulator of motor-related output for goal directed behaviors (Mallet et al., 2008; Espinosa-Parrilla et al., 2013). Contrary to the structural connectivity, the rs-fMRI connectivity for the OFC and VMPFC was higher toward the STR than toward the STN. This is somewhat puzzling as previous comparisons between DTI and rs-fMRI indicate that increased structural connectivity would predict higher functional connectivity. Whether this finding indicates that the functional connectivity between the OFC, VMPFC, and STR is driven *via* a hidden third region remains unclear (Damoiseaux and Greicius, 2009).

Overall the relative structural connectivity fingerprint of the cortex toward the STN is very similar to the STR. Compared to the other tested cortical areas, both subcortical areas have relatively low tract strengths toward the SGA, FEF, OFC, and POP. Both the SGA and OFC are thought to be involved in limbic processing. The FEF are largely governed by attentional mechanisms (Schafer and Moore, 2007), and are essential for visuospatial attention, visual awareness, and perceptual modulation such as the preparation and execution of eye movements (Bizzi, 1968) as well as smooth pursuit (MacAvoy et al., 1991) and fixation (Izawa et al., 2009). Eye movement in response to an external cue will often form the basis for action selection and appropriate motor response, and is crucial to early inhibition processes (Jantz et al., 2017). In addition it has been shown that high frequency stimulation of the STN in Parkinson’s disease patients is shown to modulate saccadic latencies (Temel et al., 2008). It was therefore surprising to also find a low functional connectivity between the STN and the FEF.

Relatedly, we found a lack of white matter connectivity between the STN, STR, and the inferior frontal gyrus pars opercularis. This was somewhat surprising given the functional significance of the inferior frontal gyrus associated with response inhibition (Aron, 2007; Swick et al., 2008; Bari and Robbins, 2013; Aron et al., 2014a,b). The low structural connectivity to the pars opercularis was complimented by relatively low functional connectivity (10 out of the 16 *t*-tests indicated substantial or more evidence in favor of lower functional connectivity for both the STN and STR).

There were also a number of cortical areas such as the CMA and SMA that compared to the other cortical areas had a stronger structural connectivity toward the subcortex. Both the CMA and SMA are thought to be crucial in voluntary based motor processes and highlight the role of the BG in action generation (Halsband et al., 1994; Shima and Tanji, 1998). The only functional connectivity that was inline with these structural connections was between the CMA and the STR.

The cortical regions were selected based on their connection with both the STN and STR as identified in NHP tracer studies. Additionally, the cortical ROIs were created using atlases that parcellated the human cortex in terms of their structural and functional homolog with NHPs using DTI (Sallet et al., 2013; Neubert et al., 2014, 2015). While there is a general agreement that major fiber tracts in DTI in NHPs correspond to the known anatomy identified with neural tracers within species, it remains difficult to identify the anatomy at the very fine fiber level with tractography (Dauguet et al., 2007; Thomas et al., 2014; Azadbakht et al., 2015; Donahue et al., 2016). Therefore, while a direct comparison between the findings of an NHP tracer study and a human neuroimaging study is challenging, previous work using DWI has shown that NHP results can be compared to humans due to similar organizational principles (Jbabdi et al., 2013). With regards to the present study, our findings are in agreement with animal-based BG models, proposing that both the STN and STR function as BG input structures and both show connections with *a priori* defined motor related, cognitive, and limbic cortical areas known to be present in NHPs. Moreover, the STN shows a higher structural connectivity with the SMA when compared with the primary motor area (M1), which is in accordance with models of action selection and inhibition within the hyperdirect pathway (Monakow et al., 1978; Haber et al., 1990; Nambu et al., 1996, 1997; Inase et al., 1999; Haynes and Haber, 2013; Feingold et al., 2015). Such a connectivity profile seems to be in line with previous work (Brunenberg et al., 2012; Lambert et al., 2012). Both the SMA and M1 connections to the STN seem to be clinically relevant as both cortico–subcortical connections are predictive for the DBS efficacy in Parkinson’s disease patients (Horn et al., 2017).

### Limitations

There are several limitations that need to be addressed. Even with a high spatial resolution of 1 mm isotropic DWI data, it remains a challenge to precisely identify where the white matter tract exactly enters the cortex resulting in the so-called “gyral biases” (Jones et al., 2013; Jbabdi et al., 2015; Reveley et al., 2015; Schilling et al., 2017). While not feasible in this project, *postmortem* MRI and histological validation of these tracts could assist in validating the *in vivo* findings presented here (Forstmann et al., 2017; Mortazavi et al., 2017). Related is the tensor model used to fit the DWI data. Here, we used a relatively simple ball-and-stick model (Behrens et al., 2007) as the acquisition parameters of the data did not lend itself for more complex models such as spherical deconvolution (Tournier et al., 2004, 2008; Dell’Acqua et al., 2012). Furthermore, the term “tract strength” should not be over interpreted as it does not quantify the actual white matter fiber number. The term tract strength here is used to index a PDF, quantifying the ratio of how many streamlines directly and continuously commence from a seed region and terminate at a target area. While this density function is a commonly used measure for inferring the strength of white matter tracts, it is not without its limitations. A related limitation is the volumetric difference of the STN and STR. While the tract strength ratio was normalized for volume and a distance correction was applied, the volume difference might still influence the result that the STR has a higher tract strength than the STN. However, this would not explain the results in which the STN tract strengths to the OFC and VMPFC were found to be stronger compared to STR. Nonetheless, the direct statistical comparisons between the STN and STR should be interpreted with caution.

A final limitation is the anatomical specificity of the cortical ROIs used in this study and the relevance for computational models. Computational models have allowed us to generate quantifiable predictions about the role of the different structures in the cortico-BG-thalamic loops (Rubchinsky et al., 2003; Brown et al., 2004; Frank, 2006; Bogacz and Gurney, 2007; Forstmann and Wagenmakers, 2015; Forstmann et al., 2017). Within a number of these models the cortex is ill-specified as a single node that can correspond to a number of distinct areas such the lateral intraparietal area (LIP), the FEF, pre-motor cortex, or simply “cortex”. Based on the current results, these models can be further refined by incorporating more precise anatomical information regarding the cortical input. A straightforward way of improving the anatomical specificity is the use of DWI and rs-fMRI to identify per participant the voxels within these relatively large cortical areas connected to the BG. These individualized cortical ROIs can be used to test correlations during task-based fMRI (e.g., Marrelec and Fransson, 2011; Zhang et al., 2012; Keuken et al., 2015). For the STN and STR, we were able to manually parcellate the structure per individual using high-resolution 7T MRI. Parcellating the entire cortex *in vivo* into the myelo- or cyto-architectonic areas per participant is, however, still very challenging (but see De Martino et al., 2014; Dinse et al., 2015; Waehnert et al., 2015). Recently, there have been a number of cortical atlases that go well beyond the cortical parcellation scheme of Brodmann (Nieuwenhuys, 2012; Nieuwenhuys et al., 2014; Glasser et al., 2016; Eickhoff et al., 2017). Such atlases entail a fine-grained parcellation of the cortex, reducing the need to use non-specific terms such as the DLPFC, which reflects a functional rather than a single anatomical defined region (Petrides and Pandya, 1999). It is, however, unclear how these recent cortical parcellations translate to the anatomical nomenclature used in the animal tracer studies.

## Conclusion

Using multimodal UHF MRI we show that compared to other tested cortical areas, the STN and STR have a relatively lower connectivity to areas thought to be involved in response inhibition and stronger connectivity to areas associated with voluntary based motor actions. Overall our results are consistent with previous literature in that the STN and STR are connected to similar cortical areas.

## Author Contributions

BI designed the study, analyzed the data, and wrote the paper. BF designed the study and wrote the paper. YT designed the study and wrote the paper. MK designed the study, analyzed the data, and wrote the paper.

## Conflict of Interest Statement

The authors declare that the research was conducted in the absence of any commercial or financial relationships that could be construed as a potential conflict of interest.

## Footnotes

1 https://www.ncbi.nlm.nih.gov/pubmed
